# A matching decomposition of the rural–urban difference in malnutrition in Malawi

**DOI:** 10.1186/s13561-014-0011-9

**Published:** 2014-09-03

**Authors:** Richard Mussa

**Affiliations:** 1 Department of Economics, Chancellor College, University of Malawi, Zomba, Malawi

**Keywords:** Matching, Decomposition, Malnutrition, Malawi

## Abstract

**Background:**

Child malnutrition remains widespread in many developing countries. Malnutrition during infancy may substantially increase vulnerability to infection and disease, and the risk of premature death. Malnutrition in children may also lead to permanent effects and to their having diminished health capital later in life as adults. These negative consequences of child malnutrition entail that the reduction of child malnutrition is vital for the social-economic development of countries. Urban children generally have better nutritional status than rural children. Malawi is no exception in this regard. The objective of this paper is to explore how much of the rural-urban nutrition gap in Malawi is explained and how much is unexplained by differences in characteristics.

**Method:**

Using data from the 2006 multiple indicator cluster survey (MICS), the paper used the Nopo decomposition method to decompose the rural-urban malnutrition gap. This nonparametric method takes into account the fact that the supports of the distributions of characteristics between the two areas can be different.

**Results:**

The results show that 90% and 89% of the stunting and underweight gaps respectively would be eliminated if there were no urban children with combinations of characteristics which positively influence child nutrition that remain entirely unmatched by rural children. Further to that, 4% and 6% of the stunting and underweight gaps respectively would disappear if there were no rural children with combinations of characteristics which negatively affect child nutrition that remain entirely unmatched by urban children.

**Conclusions:**

These findings suggest that the characteristics which negatively affect child nutrition in rural areas play a small role in the gap, and that most of the gap is largely due to the favourable characteristics such as better parental education and better household economic status among others that urban children have. The findings imply that in order to reduce the malnutrition gap policy interventions should focus more on ensuring that the favourable characteristics that urban children have such as better parental education, better household economic status among others are also available to rural children.

## Background

Child malnutrition remains widespread in many developing countries. There is ample evidence of the adverse economic and social consequences of child malnutrition. Malnutrition during infancy may substantially increase vulnerability to infection and disease, and the risk of premature death. Malnutrition in children may also lead to permanent effects and to their having diminished health capital later in life as adults. For instance, Alderman et al. [1] find that improvements in nutrition in pre-schoolers are associated with increased height as a young adult, and the number of grades of schooling completed. Case and Paxson [2] argue that the relationship between early-life nutritional deprivation and poor educational and socioeconomic outcomes as adults is both direct and indirect.

The direct channel works through impairments of cognitive ability due to early-life malnutrition that harms school success and, subsequently, labor market outcomes. The indirect channel is through early life malnutrition which translates into poor child health which in turn reduces both school attendance and attainment. This in turn worsens adult socioeconomic outcomes. Besides, early childhood malnutrition may lead to delayed school enrolment [3]–[5]. These negative consequences of child malnutrition entail that the reduction of child malnutrition is vital for the social-economic development of countries.

Urban children generally have better nutritional status than rural children [6],[7]. Malawi is no exception with regard to the rural–urban malnutrition gap. The prevalence of stunting and underweight is higher in rural Malawi. As we show in more detail in Malnutrition situation in rural and urban Malawi, data from the 2006 Multiple Indicator Cluster Survey (MICS) indicates that about 66% of urban children are mildly stunted compared to 74% in rural areas. Severe stunting is higher in rural areas with about 19% severely stunted, compared to 13% in urban areas. Almost one in five children under five in rural Malawi are moderately underweight (19%) and 3% are severely underweight. The corresponding figures for urban children are lower with 15% moderately underweight, and 2% severely underweight. This nutritional advantage that urban children enjoy entails that the aforesaid negative consequences of child malnutrition may be more pronounced in rural areas than urban areas.

Ruel et al. [8] argue that the nutritional difference between urban and rural areas is due primarily to a number of phenomena that are unique to or are exacerbated by urban living. Urban areas in relation to rural areas have a unique set of characteristics which are detrimental to child nutrition such as; greater dependence on cash income, the greater exposure to environmental contamination; greater involvement of women in income-generating activities outside the home; smaller family size and weaker social and family networks which may affect the availability of childcare. The beneficial characteristics include; greater availability of food, housing arrangements, health services and greater availability of employment opportunities. In addition, services such as electricity, water, and sanitation are on average more readily available than in rural areas. Some studies have focused on the differences in characteristics to explain the rural–urban gap in malnutrition [6],[7]. This difference in attributes may however only explain a part of the gap as some of it may be unexplained due to differences in the returns to those attributes. An equalization of the characteristics between rural and urban areas does not necessarily mean that the malnutrition gap would disappear.

With this background in mind, this paper employs the Nopo [9] decomposition method to explore how much of the rural–urban nutrition gap in Malawi is explained and how much is unexplained by differences in characteristics. To the best of our knowledge this represents the first time the Nopo decomposition has been applied to a health issue. The decomposition ensures that rural children are matched with comparable urban children.

The Nopo decomposition offers a more precise picture of where policies and programmes should target to reduce the malnutrition gap. First, it indicates part of the gap that would vanish if unmatched urban children had the same nutritional levels, on average, as their matched urban counterparts. Second, it shows how much of the rural–urban nutritional difference would be eliminated if unmatched rural children had the same nutritional levels, on average, as their matched rural counterparts. Third, it gives part of gap attributable to differences in covariates (but over the common support). Finally, it also gives the part of the gap which is unexplained by the differences in characteristics.

## Malnutrition situation in rural and urban Malawi

To assess child nutritional status we use three anthropometric indicators, the height for Age z-scores (HAZ), the weight for Age z-scores (WAZ), and the weight for height z-scores (WHZ). Following a common empirical regularity, we use the U.S National Center for Health Statistics (NCHS) as recommended by the World Health Organization (WHO) as a reference population. The three indicators measure different dimensions of child nutrition status. The HAZ measures stunting, WAZ assesses underweight, and finally the WHZ determines wasting. The most commonly used cut-off to define abnormal anthropometry is a value of −2, that is, two standard deviations below the reference median, irrespective of the indicator used. Specifically; HAZ, WAZ, and WHZ values less than or equal to −2 indicate stunting, underweight, and wasting respectively. The WHO also has a more general malnutrition classification that distinguishes between mild (z-score ≤ −1), moderate (z-score ≤ −2), and severe malnutrition (z-score ≤ −3) [10].

Table 1 reports the percentages of mildly, moderately, and severely malnourished children in rural and urban Malawi. Means of the three nutrition indicators are also displayed. The results show noticeable rural–urban differences in the proportion of children who are malnourished. About 66% of urban children are mildly stunted compared to 74% in rural areas. Severe stunting is higher in rural areas with about 19% severely stunted, compared to 13% in urban areas. Almost one in five children under five in rural Malawi are moderately underweight (19%) and 3% are severely underweight. The corresponding figures for urban children are lower with 15% moderately underweight, and 2% severely underweight.

**Table 1:** Percentage of under-five children who are malnourished

	**Rural**			**Urban**		
	**HAZ (stunting)**	**WAZ (underweight)**	**WHZ (wasting)**	**HAZ (stunting)**	**WAZ (underweight)**	**WHZ (wasting)**
Mild	74.0	54.3	16.9	65.7	47.1	15.9
Moderate	46.2	19.2	3.87	35.4	14.7	4.09
Severe	19.2	3.31	0.81	12.8	2.17	1.01
Mean	−1.799	−1.050	0.091	−1.468 1.468***	−0.844***	0.101

*Notes:* own computations from MICS data. Malnutrition is classified as follows; mild (z-score ≤ −1), moderate (z-score ≤ −2), and severe malnutrition (z-score ≤ −3). We test the hypothesis that the mean of a malnutrition indicator in urban areas is greater than (that is less negative) that of rural areas. The significance asterisks are defined as: *p < 0.10, **p < 0.05, ***p < 0.01.

Looking at wasting, the rural–urban difference is mixed as it depends on the extent of wasting. Moderate wasting stands at 4% for urban children, and 3.8% for rural children. Severe wasting is slightly higher in urban areas than in rural areas, while the reverse holds for mild wasting. The means of the indicators (last row in Table 1) tell a similar story to the malnutrition prevalence rates; which is that stunting and underweight are worse in rural areas than in urban areas, and with the means all positive, wasting is not a problem. The results also indicate that mean differences are statistically significant for stunting and underweight only. The WHZ, and to some extent the WAZ are more prone to acute episodes of stress occurring at or around the time of measurement [11]. They are thus short term indicators of malnutrition. The results seem to suggest that under five children in rural areas compared to their urban counterparts fare poorly in terms of long term indicators of malnutrition but the differential is small and mixed when looked at in terms of short term indicators.

The finding that in Malawi there is little if at all any difference between rural and urban areas in terms of wasting is consistent with other studies which find very small rural–urban differences and even in a few cases, slightly higher wasting in urban areas (for example [6]). This lack of difference seems to persist overtime, as Smith et al. [7] using 1992 data find no statistically significant difference in the means of WHZ between rural and urban children in Malawi. Consequently, this paper focuses on the examination of the difference between rural and urban children with respect to stunting and underweight only.

## Methods

This section begins with a discussion of the matching approach which we use in the empirical analysis to explore the rural–urban gap in stunting and underweight. This is followed by a presentation of the explanatory variables used.

### Description of the matching approach

As mentioned earlier, the paper adopts a decomposition approach proposed by Nopo [9]. It is an extension of the standard Blinder-Oaxaca decomposition, independently proposed by Oaxaca [12] and Blinder [13]. It is better than the Blinder-Oaxaca decomposition as it addresses two limitations of the Blinder-Oaxaca decomposition.

The first limitation is that it is fully parametric since one is required to estimate a linear regression model for malnutrition. This imposes a restriction on the functional relationship between malnutrition and its determinants. The second and perhaps more important limitation is that it ignores the common support problem by estimating malnutrition equations for all rural children and all urban children without restricting the comparison only to those children with comparable characteristics. The decomposition is thus based on an out-of-support assumption. Individual child characteristics in rural and urban areas may not necessarily overlap. There may be a mismatch in child characteristics between rural and urban areas. For certain combinations of child characteristics it may be possible to find urban children, but not rural children (for example mothers with tertiary education in urban areas) while there are also combinations of characteristics for which it is possible to find rural children, but not urban children (for example drinking water from wells in rural areas).

The Nopo decomposition is a fully nonparametric method as it does not require the estimation of a linear malnutrition regression model. Critically, it does not make the out-of-support assumption as the counterfactual mean malnutrition level is simulated only for the common support. In order to construct the counterfactual mean malnutrition, Nopo [9] uses an exact covariate matching procedure which selects two sub-samples of rural and urban children with comparable characteristics. We now discuss the matching procedure.

Let *H* denote the child nutritional status indicator (HAZ, WAZ), and *X* the vector of individual characteristics which determine child nutrition. Furthermore, let *g*^*u*^(*x*) = *E*(*H*|*X* = *x*, *u*) denote the mean of the child nutritional status indicator for urban areas, with characteristics *x*, *F*^*u*^(*x*) the cumulative distribution function of individual characteristics *x* for urban areas, and *S*^*u*^ the support of the distribution of characteristics for urban areas. For rural areas, *g*^*r*^(.), *F*^*r*^(.) and *S*^*r*^ are defined in a similar manner. The average rural–urban malnutrition gap is then expressed as

(1) $$\Delta =E\left(H|u\right)-E\left(H|r\right)$$

To allow for the possibility that the support of the distribution of characteristics for urban children, *S*^*u*^, is different than the support of the distribution of characteristics for rural children, *S*^*r*^, the mean malnutrition level for each group is further subdivided over its respective domain into two parts: one on the intersection of the supports, *S* = *S*^*u*^ ∩ *S*^*r*^ and one out of the common support, $\bar{S}$. The mean malnutrition level for urban children then becomes

(2) $$E\left(H|u\right)=E_{s}\left(H|u\right)\theta_{s|u}+E\left(H|u\right)$$

Where; *θ*_*S*|*u*_ = *θ*(*X* ∈ *S*|*u*) = ∫ _*s*_*dF*^*u*^(*x*) is the probability measure of the set *S* under the distribution *dF*^*u*^(.). Noting that $\theta_{\bar{S}|u}=\theta \left(X\in \bar{S}|u\right)=1-\theta_{S|u}$, equation (2) can be rewritten as:

(3) $$E\left(H|u\right)=\theta_{\bar{S|}u}\left[E_{\bar{S}}\left(H|u\right)-E_{s}\left(H|u\right)\right]+E_{s}\left(H|u\right)$$

The corresponding mean malnutrition level for rural children is similarly derived to get

(4) $$E\left(H|r\right)=\theta_{\bar{S}\left|r\right)}\left[E_{\bar{S}}\left(H|r\right)-E_{s}\left(H|r\right)\right]+E_{s}\left(H|r\right)$$

Substituting the mean malnutrition level for urban children (equation (2)) and the mean malnutrition level for rural children (equation (4)) into the average child malnutrition gap (equation (1)) we get

(5) $$\begin{array}{l} \Delta =E\left(\left(H\right|u\right)-E\left(\left(H\right|r\right)\\ =\underset{I}{\underbrace{\left[E_{S}\left(\left(H\right|u\right)-E_{S}\left(\left(H\right|r\right)\right)}}+\underset{\mathrm{II}}{\underbrace{\theta_{\bar{S}\left|u\right)}\left[E_{\bar{S}}\left(H\left|u\right)\right)\right]-E_{S}\left(H\left|u\right)\right)}}\\ \;+\underset{\mathrm{III}}{\underbrace{{\theta_{\bar{S}}}_{\left|r\right)}\left[E_{\bar{S}}\left(\left(H\right|r\right)-E_{S}\left(\left(H\right|r\right)\right]}} \end{array}$$

Part I of this expression measures the rural–urban difference in average child malnutrition over the common support only, while parts II and III capture the average child malnutrition difference between urban and rural children respectively in and out-of-the support.

Part I can further be decomposed by adding and subtracting the counterfactual mean malnutrition $\int_{S}g^{u}\left(x\right)dF_{s}^{r}\left(x\right)$, with $dF_{s}^{r}\left(x\right)$ the density of characteristics in the subpopulation of rural children in the common support. As indicated earlier, the counterfactual mean malnutrition for rural children (urban children) represents the average malnutrition level for rural children (urban children) if they were urban children (rural children). Part I then becomes;

(6) $$\begin{array}{l} E_{s}\left(H\left|u\right)\right)-E_{s}(H\left|r\right))=\int_{S}g^{u}(x)dF_{s}^{u}(x)-\int_{S}g^{r}(x)dF_{s}^{r}(x)\\ \;=\int_{S}g^{u}\left(x\right)[dF_{s}^{u}(x)-dF_{s}^{r}(x)]+\int_{S}\left[g^{u}\left(x\right)-g^{r}\left(x\right)]dF_{s}^{r}(x\right) \end{array}$$

Similar to the standard Blinder-Oaxaca decomposition, the first and the second parts of equation (6) capture the characteristic and the coefficient effects of the rural–urban malnutrition gap, but now on the common support only. Putting everything together, the overall malnutrition gap, ∆ is broken into four additive components as follows

(7) $$\Delta =\Delta_{u}+\Delta_{x}+\Delta_{o}+\Delta_{r}$$

Where;

(8) $$\begin{array}{l} \Delta_{u}=\theta_{\bar{S}|u}[E_{\bar{S}}\left(\mathrm{H|u}\right)-E_{S}[E(\mathrm{Hu})]\\ \Delta_{x}=\int_{S}g^{u}\left(x\right)[dF_{S}^{u}(x)-dF_{S}^{r}(x)]\\ \Delta_{o}=\int_{S}[g^{u}\left(x\right)-g^{r}(x)]dF_{S}^{r}(x)\\ \Delta_{r}=\theta_{\bar{S}|r}[E_{\bar{S}}\left(\mathrm{H|r}\right)-E_{S}\left(\mathrm{H|r}\right)] \end{array}$$

The component ∆_*u*_ represents part of the gap which can be explained by differences between those urban children whose characteristics can be matched to rural children’s characteristics and those who remain unmatched. Thus, this is part of the rural–urban nutritional difference that would be eliminated if there were no urban children with combinations of characteristics *X* that remain entirely unmatched by rural children, or alternatively if these unmatched urban children had the same nutritional levels, on average, as their matched urban counterparts. For instance urban children may have easy access to clean drinking water and medical care which rural children may not. This component may therefore explain the rural–urban differences in nutrition which arise from the fact that some characteristics which influence positively child nutrition may be available to urban children only.

The component ∆_*r*_ is interpreted in a similar way between matched and unmatched rural children. For this component, there are no urban children who have the same characteristics as rural children. It is part of the gap which would disappear if all rural children had at least one possible combination of the set of characteristics *X* that the population of urban children have, or alternatively, if these unmatched rural children had the same nutritional levels, on average, as their matched rural counterparts. For instance, rural children may drink poor quality water from wells which may not be the case for most urban children. This component sheds light on the rural–urban gap in malnutrition attributable to the fact that some characteristics which negatively affect child nutrition may be available to rural children only.

The components ∆_*x*_ and ∆_*o*_ are similar to the standard Blinder-Oaxaca decomposition’s characteristic effect and coefficient effect except that this is over the common support. The component ∆_*x*_ captures part of rural–urban malnutrition gap attributable to differences in covariates (but over the common support). For example, rural and urban mothers may have secondary or tertiary education, but urban mothers are more represented in this category than rural mothers. Thus, ∆_*x*_ measures the decrease in the malnutrition gap if the distributions of characteristics of rural children and urban children are equalized over the common support. The component ∆_*o*_ is the residual part of the malnutrition gap. It is part of the gap which is unexplained by the differences in characteristics. It the nutritional gap which remains even if urban and rural children had the same characteristics over the common support. In the standard linear Blinder-Oaxaca decomposition the characteristic effect is Δ_*u*_ + Δ_*x*_ + Δ_*r*_ and the coefficient effect is ∆_*o*_.

The Nopo decomposition uses an exact matching algorithm to estimate the counterfactual mean malnutrition as well as the four components. Exact matching means that a rural child is matched whenever we find an identical urban child in terms of *X*. The treatment variable is area of residence, rural vs. urban. The algorithm involves four steps as summarized as below.

 Step 1: For each rural child in the sample, do steps 2 and 3.

 Step 2: Select all observations from the sub-sample of urban children who have the same characteristics as the rural children of step 1. Do not remove these selected observations such that they can be used again. Denote these urban children as matched. If no observations are selected in this step, denote the rural children chosen in step 1 as unmatched, otherwise as matched.

 Step 3: Compute the counterfactual mean malnutrition level of the rural children selected in step 1 as the weighted average malnutrition level of the urban children selected in step 2.

 Step 4: Compute Δ_*u*_, Δ_*x*_, Δ_*r*_ and ∆_*o*_ using the actual malnutrition variable, the new synthetic malnutrition variable and the “match” dummy variable, coded as 1 if a rural child (urban child) is matched to an urban child (rural child).

### Variables used

As indicated earlier, we have two dependent variables namely; the HAZ and the WAZ. In terms of independent variables, we have three categories of variables; child level variables, household level variables, and regional level variables. Child level variables included are; a child’s age in months and its square to capture possible non linearities, sex of the child, and the status of being a twin, as twins frequently show lower birth weight [14]. We also control for the child’s birth order. At the household level we have the age difference between mother and father to capture the bargaining position of the mother. According to the bargaining literature on household decisions, bargaining status could influence those resources that the mother may receive for herself as well as for her child, possibly leading to adverse nutrition consequences [15].

The economic status of a child’s household is known to be a strong determinant of her or his nutritional status. Poor households and individuals often have low access to food, a necessary condition for food security. They also may have inadequate resources for care, and may not be able to utilize (or contribute to the creation of) resources for health on a sustainable basis [7]. We measure household economic status by using a wealth index, and the households are categorized into five groups; poor, middle, richer, and richest. The poorest group is the base category. Parental education is included as a three class dummy variable indicating whether the mother/father has primary schooling, or has secondary or more education, no education for mothers and fathers represent the control group.

We include a dummy to capture whether the mother was a teenager at the birth of the child. Children of mothers who were teenagers when giving birth may have lower nutritional status [14]. We also include ethnicity of the household (chewa, lomwe, yao, ngoni, tumbuka. Other tribes represent the excluded category. The religion of the family is also included classified as follows; protestant, muslim, catholic, with other religions representing the excluded category. Finally, at the regional level we control for region effects, by including dummies north and centre, with south as the base.

### Data

This paper uses data from the 2006 Multiple Indicator Cluster Survey (MICS) which was conducted by Malawi’s National Statistical Office. The MICS are a survey program developed by the United Nations Children's Fund to provide internationally comparable, statistically rigorous data on the situation of children and women. It therefore provides good quality and reliable data. In terms of quality of data collection, this was ensured through continual monthly monitoring of fieldwork by field staff. Further to this, all questionnaires were double entered and internal consistency checks were performed.

The main objective of the MICS was to obtain estimates at district level on the key indicators related to the wellbeing of children and women. The survey covers 26 districts with 2 districts, Likoma and Neno merged with other districts. From each district a total of 1200 households were sampled. Two-stage sampling was used to select the 1200 households. In the first stage in each district, 40 census enumeration areas (clusters) were selected. In the second stage a household listing was performed within the cluster and a systematic sample of 30 households was drawn to obtain 1,200 households per district.

A total of 31200 households were selected in 1,040 clusters. This makes the MICS one of the largest nationally representative household surveys in Malawi. Besides, one of the challenges faced by policy makers and programme managers in Malawi is the lack of sub-national data, and MICS is the only survey which attempts to address this problem. Since the survey’s main focus is on the situation of children and women, it collected information on; children under five, all women aged 15–49 years, and men aged 15–49 in every third household selected. Information on child anthropometrics was collected, and this is of interest to this paper as it focuses on child malnutrition. We have a total of 53879 under five children in the sample. This total sample is subdivided into 48454 under five children from rural areas, representing 90% of the sample, and 5425 from urban areas, constituting 10% of the sample.

## Results

This section presents descriptives of explanatory variables used, means for a selection of variables for unmatched and common support samples, and finally, we look at the Nopo and Blinder-Oaxaca decomposition results.

### Descriptive statistics of explanatory variables

Table 2 presents descriptive statistics of explanatory variables used for rural and urban under five children. The statistics as expected show that there are differences in the attributes of rural and urban children. There are slightly more boys in urban areas than rural areas. In terms of household economic status, there are more rich households in urban areas than in rural areas. Close to 60% of urban households fall in the richest category as compared to 11% of households in rural areas. The mean age difference for rural mothers is higher than that of urban mothers suggesting that they have a weaker bargaining position relative to their urban counterparts.

**Table 2:** Descriptive statistics of variables

**Variable**	**Rural**		**Urban**	
	**Mean**	**Std Dev**	**Mean**	**Std Dev**
Child Characteristics				
Boy	0.496	0.499	0.518**	0.499
Twin	0.029	0.169	0.026	0.156
Age of child (in months)	27.019	19.210	28.052***	18.929
Age of child squared	1099.083	1166.593	1145.205***	114.887
Birth order	4.531	2.518	3.755***	2.305
Household economic status				
Poorest	0.248	0.432	0.049***	0.216
Poor	0.228	0.420	0.068***	0.252
Middle	0.221	0.415	0.1174***	0.322
Richer	0.191	0.393	0.166***	0.372
Richest	0.111	0.314	0.599***	0.490
Mother Characteristics				
Age difference	7.209	10.190	6.856*	8.877
Teen age mother	0.197	0.295	0.126***	0.332
No education	0.293	0.455	0.142***	0.349
Primary education	0.685	0.465	0.684	0.465
Secondary education +	0.071	0.257	0.287***	0.452
Father Characteristics				
No education	0.192	0.394	0.091***	0.288
Primary education	0.684	0.465	0.502***	0.500
Secondary education +	0.139	0.347	0.443***	0.497
Religion				
Protestant	0.637	0.481	0.656**	0.475
Muslim	0.123	0.329	0.139**	0.346
Catholic	0.197	0.397	0.208*	0.406
Other	0.039	0.194	0.021***	0.145
Ethnicity				
Chewa	0.335	0.472	0.216***	0.411
Lomwe	0.159	0.366	0.159	0.365
Yao	0.118	0.323	0.137***	0.343
Ngoni	0.117	0.322	0.127*	0.333
Tumbuka	0.113	0.316	0.215***	0.411
Other	0.187	0.390	0.206***	0.405
Region				
North	0.199	0.399	0.295***	0.456
Centre	0.382	0.486	0.300***	0.458
South	0.418	0.493	0.405	0.490
Observations	48454		5425	
Share (%)	90		10	

*Notes* We test the null hypothesis of no rural–urban mean difference in the regressors. The significance asterisks are defined as: *p < 0.10, **p < 0.05, ***p < 0.01.

The proportion of teen age mothers is higher in rural areas than in urban areas. The results show that about 20% of mothers in rural areas had a child as teen agers as compared to about 13% in urban areas. Regarding parental education, the results indicate that urban 29% of urban children have mothers with secondary education or more, while only 7% of mothers have secondary education or more in rural areas. The education gap is even more pronounced for fathers, with 44% and 14% having secondary education or more in urban and rural areas respectively.

### Child characteristics and malnutrition rates in and out of the common support

Table 3 reports means of selected variables for unmatched rural, unmatched urban and common support (matched) children. This is useful as it gives us a sense of how the characteristics differ for the matched and unmatched children. Overall, of the 53879 children in the sample, 41847 rural children representing 80% are unmatched with urban children, 4536 of urban children, representing 8% have no match in rural areas, and finally, 14% of urban and rural children are matched. Owing to the fact that we are matching on the same independent variables, the results for both the HAZ and the WAZ are basically identical, and they show that there are noticeable differences in the three samples with respect to child attributes.

**Table 3:** Means for matched, unmatched, and common support samples

**Variable**	**HAZ and WAZ**		
	**Unmatched rural**	**Unmatched urban**	**Common support**
HAZ	−1.802	−1.410	−1.783
WAZ	−1.049	−0.798	−1.068
Mild stunting	0.745	0.644	0.729
Moderate stunting	0.466	0.338	0.458
Severe stunting	0.192	0.127	0.191
Mild underweight	0.543	0.456	0.570
Moderate underweight	0.196	0.144	0.188
Severe underweight	0.033	0.019	0.042
Poorest	0.238	0.046	0.281
Poor	0.224	0.066	0.235
Middle	0.230	0.106	0.159
Richer	0.192	0.163	0.180
Richest	0.113	0.617	0.142
Age difference	7.218	6.927	4.419
Teen age mother	0.086	0.109	0.171
No education	0.289	0.137	0.302
Primary education	0.682	0.676	0.703
Secondary education +	0.066	0.276	0.130
No education	0.172	0.082	0.295
Primary education	0.693	0.485	0.617
Secondary education +	0.149	0.470	0.102
Observations	41847	4536	7496
Share (%)	77.67	8.42	13.91

Generally, unmatched urban children have favourable characteristics compared to unmatched rural children, and matched children. In addition, unmatched rural children and matched children have fairly similar attributes. Looking at household economic status, the results indicate that the majority, 67%, of unmatched urban children belong to households in the richest category. In stark contrast, 11% and 14% of unmatched rural children, and matched children respectively belong to households in the richest category.

Turning to parental education, the results show that 28% and 47% of mothers and fathers respectively of unmatched urban children have secondary education or more. These are higher than corresponding percentages for unmatched rural children and matched children. Besides, relative to unmatched urban children, a larger percentage of unmatched rural children and those who are matched have parents who have no education. The similarity of characteristics between unmatched rural children and matched children offers some insight into the nature of matched sample, which is that rural and urban children are matched on a set of characteristics which are detrimental to their nutritional wellbeing rather than beneficial ones. This therefore suggests low end matching.

**Figure 1:**
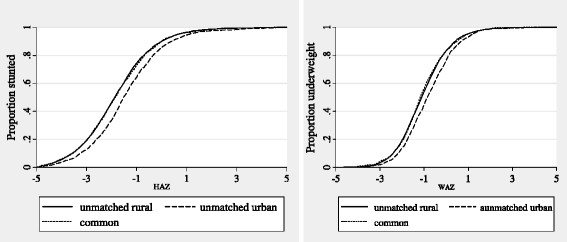
CDFs for matched, unmatched, and common support samples.

We next take a look at the differences in prevalence rates of stunting and underweight for children in the unmatched rural, unmatched urban and common support samples. We do this by using cumulative density functions (CDFs). The CDFs basically show whether or not the distribution of a malnutrition variable in one area first order stochastically dominates that of another area. A CDF for area A which is everywhere below that of area B means that area B has a higher proportion of malnourished children than area A irrespective of cut-off point chosen. That is, area A first order stochastically dominates area B. Figure 1 shows the CDFs for the three samples, and for the HAZ and WAZ. The CDFs for the two nutrition indicators are largely similar.

Looking at the HAZ for instance, the CDFs for the unmatched rural children and matched children are observationally indistinguishable, with no discernible pattern of dominance by one sample over the other. This suggests that the prevalence of stunting in the two samples is similar. The same can be said of the proportion of underweight children in the two samples. This lack of difference in prevalence rates between unmatched rural and matched samples supports our earlier finding that the matched samples are matched on a set of unfavorable characteristics which are harmful to their nutritional status.

Interestingly, for both the HAZ and the WAZ, the CDFs for unmatched urban children are below those for unmatched rural children and matched children. Thus, the sample of unmatched urban children first order stochastically dominates the other two samples. This means that regardless of malnutrition cut-off point used, the proportion of stunted and underweight children is the lowest for unmatched urban children. This may be a reflection of the fact seen earlier that unmatched urban children have favourable characteristics which positively affect their nutritional status.

Taking cognizance of the fact that the choice of the variables included in the matching procedure is somewhat arbitrary but could have a big impact on the percentage of rural or urban children that are matched, we conducted a sensitivity analysis of the percentage of children who are in the common support. The sensitivity analysis seeks to establish whether or not some variables have the largest impact on the percentage of children who are matched and whether different combinations of variables produce substantively similar results. The variables were grouped into three categories namely: child characteristics, parental characteristics, and household characteristics. Compared to the case where all the variables are used, the results of this sensitivity analysis indicate that the quality of the matched sample does not significantly differ across the different categories of characteristics: the matched percentages are 14.7% with child characteristics only, 12.3% with parental characteristics only, and 16.3% with household characteristics only. All this implies that the conclusions of this paper are insensitive to choice of matching covariates employed.

### Matching decomposition results

Table 4 contains Nopo decomposition results for the rural–urban malnutrition gap. As indicated earlier, the average malnutrition gap is the difference between averages of the nutrition indicators for urban and rural children. Thus, a positive gap means that rural children are on average worse off relative to urban children. The results for the HAZ and the WAZ are qualitatively similar. For the HAZ, the average malnutrition gap of 0.331 is decomposed as: 91% is explained by differences in characteristics outside the common supports of urban children, 4% is explained by differences in characteristics outside the common supports of rural children, −1% is explained by differences in the distributions of individual characteristics within the common support, and the remaining 5% is the part of the gap which is unexplained by differences in characteristics between the two areas.

**Table 4:** Nopo decomposition of the rural–urban malnutrition gap

	**HAZ**		**WAZ**	
	**Coefficient**	**Percent of ∆**	**Coefficient**	**Percent of ∆**
**Raw gap, ∆**	**0.331**	**100.00**	**0.206**	**100.0**
Of which:				
∆_*u*_	0.300	90.77	0.184	89.18
∆_*r*_	0.014	4.35	0.012	5.90
∆_*X*_	−0.004	−1.09	−0.004	−2.13
∆_*o*_	0.018	5.44	0.014	7.01

For the WAZ, the average malnutrition gap of 0.206 is decomposed as: 89% is attributable to differences in characteristics outside the common supports of urban children, 6% arises from differences in characteristics outside the common supports of rural children, −2% is due to differences in the distributions of individual characteristics within the common support, and the remaining 7% is the unexplained part of the gap.

### Matching and blinder-oaxaca compared

The Nopo decomposition results we have just seen are based on the common support assumption which ensures that ‘like is compared with like’, and ignoring this may lead to misleading results since children are compared though they are not comparable. In Table 5 we present Blinder-Oaxaca decomposition results. These results give us a sense of the effect of ignoring the common support assumption in decomposing the nutrition gap. The results for both the HAZ and the WAZ are generally similar to the Nopo decomposition results; to the extent that both methods suggest that the characteristic effect is what drives the rural–urban malnutrition gap.

**Table 5:** Blinder-Oaxaca decomposition of the rural–urban malnutrition gap

	**HAZ**		**WAZ**	
	**Coefficient**	**Percent of ∆**	**Coefficient**	**Percent of ∆**
**Raw gap, ∆**	**.379**	**100.00**	**.248**	**100.00**
Of which:				
Explained	.234	61.7	.164	66.3
Unexplained	.145	38.3	.083	33.7

A notable difference between the two results is that the Blinder-Oaxaca decomposition overestimates the unexplained part of the gap (the coefficient effect) and underestimates the explained part of the gap (the characteristic effect). For instance, for the HAZ, 62% of the gap is the characteristic effect, while 38% is the coefficient effect. This difference between the two decompositions could not necessarily be due to the fact that the Blinder-Oaxaca decomposition ignores the common support assumption; it could well be that by assuming linearity we are committing a functional form specification error.

To check if this is the case, we restricted the Blinder-Oaxaca decomposition to the matched sample only. If the linear specification of the nutrition regressions on the common support is correct, then we should have similar results to those obtained after matching. We find that restricting the Blinder-Oaxaca decomposition to matched samples gives a characteristic effect of 92% and 86% for the HAZ and the WAZ respectively. On the strength of these results, we can conclude that ignoring the common support assumption is behind the observed differences between the two decomposition methods.

## Discussion

These results suggest that differences in characteristics (the characteristic effect) rather than differences in the returns to those characteristics (the coefficient effect) are the major driver of stunting and underweight gaps between the two areas. More precisely, 90% and 89% of the stunting and underweight gaps respectively would be eliminated if there were no urban children with combinations of characteristics which positively influence child nutrition that remain entirely unmatched by rural children. Further to that, 4% and 6% of the stunting and underweight gaps respectively would disappear if there were no rural children with combinations of characteristics which negatively affect child nutrition that remain entirely unmatched by urban children.

These findings suggest that the characteristics which are disadvantageous to child nutrition in rural areas play a small role in the gap, and that most of the gap is largely due to the favourable characteristics that urban children have. Although, one cannot attach a causal interpretation, the findings suggest that in order to reduce the malnutrition gap attention should focus more on ensuring that the favourable characteristics that urban children have such as better parental education, better household economic status among others are also available to rural children. Besides, differences in the distribution of characteristics for matched rural and urban children have a negligible effect on the gap. Interestingly, the results indicate that if the distributions of characteristics of matched rural children and matched urban children were to be equalized this would worsen the gap. This is perhaps a reflection of the fact discussed earlier that the matched sample is matched on a low end of characteristics which exacerbate the gap instead of reducing it.

A comparison of the results from the Nopo decomposition and the Blinder-Oaxaca decomposition reveal some interesting advantages that the Nopo decomposition has. Specifically, the Blinder-Oaxaca decomposition overestimates the unexplained part of the gap (the coefficient effect) and underestimates the explained part of the gap (the characteristic effect). Thus, by ignoring the common support problem, and therefore not comparing like with like, the Blinder-Oaxaca decomposition gives a misleading picture of the drivers of malnutrition in Malawi.

## Conclusions

The paper has looked at the rural–urban differential in child malnutrition in Malawi. Using data from the 2006 multiple indicator cluster survey (MICS), we have used a matching method to decompose the rural–urban malnutrition gap. This nonparametric method takes into account the fact that the supports of the distributions of characteristics between the two areas can be different. Matching allows the decomposition to be done over the common support, and this is important because comparisons in malnutrition are relevant only when rural families are compared to “comparable” urban families.

We use HAZ to measure stunting, and the WAZ to capture underweight. The results show that the average malnutrition gap of 0.331 for the HAZ is decomposed as: 91% is explained by differences in characteristics outside the common supports of urban children, 4% is explained by differences in characteristics outside the common supports of rural children, −1% is explained by differences in the distributions of individual characteristics within the common support, and the remaining 5% is the part of the gap which is unexplained by differences in characteristics between the two areas. A similar picture emerges for the WAZ.

The results also show that ignoring the common support assumption leads to misleading conclusions about the extent to which rural–urban gaps in malnutrition are driven by differences in characteristics or differences in coefficients. Precisely, we find that the unexplained part of the gap (the coefficient effect) is overestimated and the explained part of the gap (the characteristic effect) is underestimated.

These findings suggest that the characteristics which negatively affect child nutrition in rural areas play a small role in the gap, and that most of the gap is largely due to the favourable characteristics such as better parental education and better household economic status among others that urban children have. Without purporting to attach a causal interpretation, the findings imply that in order to reduce the malnutrition gap policy interventions should focus more on ensuring that the favourable characteristics that urban children have such as better parental education, better household economic status among others are also available to rural children. Besides, differences in the distribution of characteristics for matched rural and urban children have a negligible effect on the gap.

## Competing interests

The authors declare that they have no competing interests.
